# Hepatic falciform ligament appendagitis evaluated by ultrasound: A report of 2 cases

**DOI:** 10.1016/j.radcr.2022.08.090

**Published:** 2022-09-20

**Authors:** Daisuke Miura, Michiko Shindo, Yoshihisa Fukuda

**Affiliations:** a Department of Clinical Laboratory, Fukuoka Tokushukai Medical Center, 4-5 Sugu Kita, Kasuga-shi, Fukuoka-ken, 816-0864, Japan; b Department of Laboratory Science, Yamaguchi University Graduate School of Medicine, 1-1-1 Minami Kogushi, Ube-shi, Yamaguchi-ken, 755-8508, Japan; c Department of Radiology, Fukuoka Tokushukai Medical Center, 4-5 Sugu Kita, Kasuga-shi, Fukuoka-ken, 816-0864, Japan; d Department of Gastroenterology, Fukuoka Tokushukai Medical Center, 4-5 Sugu Kita, Kasuga-shi, Fukuoka-ken, 816-0864, Japan

**Keywords:** Ultrasound, MRI, Falciform ligament, Falciform ligament appendage torsion, Falciform ligament appendagitis, Hepatic falciform ligament appendagitis

## Abstract

Falciform ligament appendagitis is an extremely rare disorder, which is characterized by hematogenous or nonhematogenous inflammatory changes in the fat appendage that is contiguous with the falciform ligament. The imaging and clinical features of this condition are similar to those of epiploic appendagitis, especially when caused by torsion of the fatty appendage (ie, falciform ligament appendage torsion). We report 2 cases of falciform ligament appendagitis with localized epigastric pain. The ultrasound imaging features of the 2 cases presented here were an oval hyperechoic mass contiguous with the falciform ligament and increased echogenicity of the surrounding inflammatory fat. Both patients were managed conservatively with symptomatic treatment alone. Understanding the imaging features of this falciform ligament appendagitis is important, because ultrasound is often the first choice for noninvasive imaging of acute abdomen. As there is limited detailed literature on falciform ligament appendagitis comparing high-frequency linear probes with CT and MRI, we consider this case report to add valuable information on this poorly reported condition.

## Introduction

The first diagnosis of falciform ligament appendage torsion (FLAT) using CT and ultrasound was reported by Coulier et al. in 2001 [1]. Falciform ligament appendagitis (FLA) is an inflammation of the fatty appendage that is contiguous with the falciform ligament and includes FLAT. Intra-abdominal focal fat infarction (IFFI) is the general term that encompasses adipose tissue diseases, such as epiploic appendicitis and omental infarction, and FLA can be broadly included in IFFI. In this report, we described 2 FLA cases that were observed in detail on high-frequency linear ultrasound probe and compared with CT and MRI.

## Case report

**Fig. 1:**
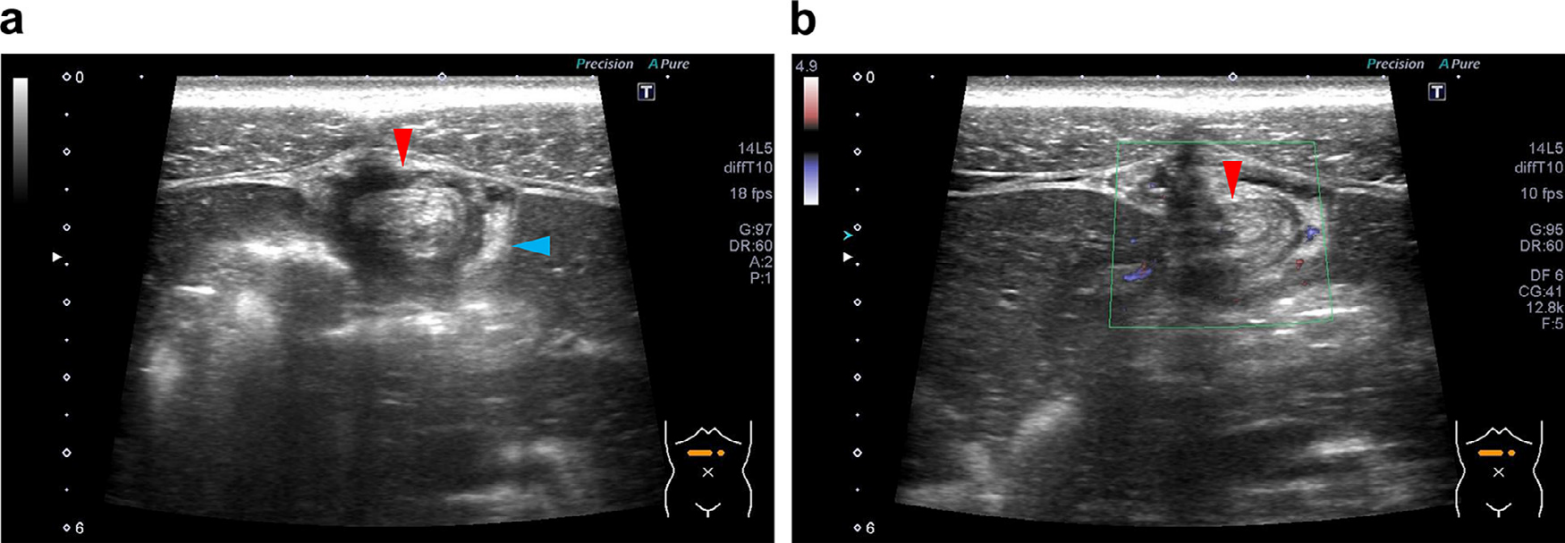
Ultrasound findings in Case 1. (A) There is a 15 × 10-mm oval-shaped hyperechoic mass adjacent to the falciform ligament with a peripherally located hypoechoic rim (red arrow head) and hyperechogenic surrounding fat (blue arrow head). (B) No internal vascularity signal is seen on Doppler evaluation.

**Fig. 2:**
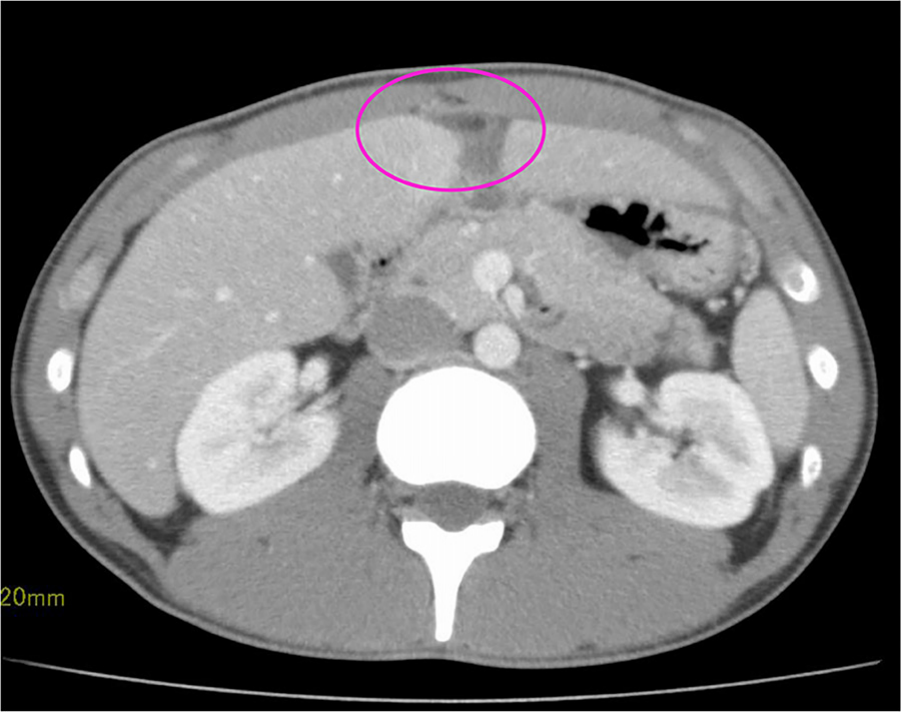
Contrast-enhanced computed tomography findings in Case 1. Thickening of the hepatic round ligament and increased surrounding fat concentration are seen (circle).

**Case 1:** A 25-year-old man was referred to our emergency room with persistent epigastric pain. His body mass index (BMI) was 18.5 kg/m^2^. Ultrasound and abdominal X-ray done at local clinic a few hours prior did not identify the cause. Palpation revealed localized tenderness on the epigastric area without guarding or rebound tenderness. Blood tests revealed a white blood cell (WBC) count of 10,720/ μL and a C-reactive protein (CRP) value of 0.13 mg/dL. For the ultrasound examinations, we used Aplio 400 (Canon Medical Systems, Tochigi, Japan) with a 14L5 linear probe set at 10-MHz frequency. Ultrasound revealed an oval-shaped hyperechoic mass, which had a hypoechoic rim and was adjacent to the falciform ligament, and increased echogenicity and disturbance of the surrounding fat (Fig. 1). Color Doppler revealed absent blood flow signal inside the mass, possibly reflecting infarction. These ultrasound findings suggested ischemic or inflammatory changes in the fatty appendage that was contiguous with the falciform ligament. On the same day, contrast-enhanced CT showed thickening of the hepatic round ligament and increased concentration of surrounding fat, suggesting edema and inflammatory changes (Fig. 2). The oval mass that was noted on ultrasound was not evident, but the inflammation of the hepatic round ligament/falciform ligament was noted. Based on both ultrasound and CT findings, we strongly suspected inflammatory/edematous changes in the fatty appendage attached to the falciform ligament, diagnosed FLA. The symptoms resolved the next day, and the patient was discharged with a prescription of a proton pump inhibitor and hyoscine-N-butylbromide. He was instructed to follow-up if symptoms persisted, but has not done so thereafter. This rapid clinical course suggests that the fatty appendage may have had a transient torsion.

**Fig. 3:**
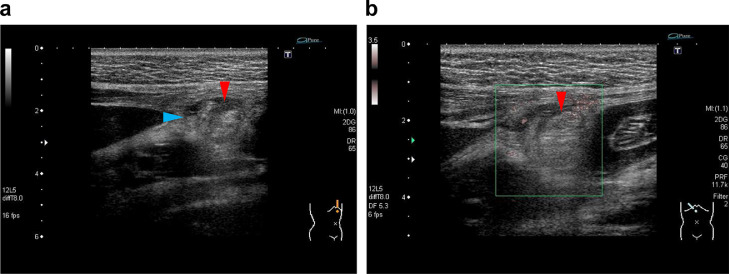
Ultrasound findings in Case 2. (A) There is a 17 × 15-mm oval-shaped hyperechoic mass adjacent to the falciform ligament with a peripherally located hypoechoic rim (red arrow head) and hyperechoic surrounding fat (blue arrow head). (b) No internal vascularity signal is seen on Doppler evaluation.

**Fig. 4:**
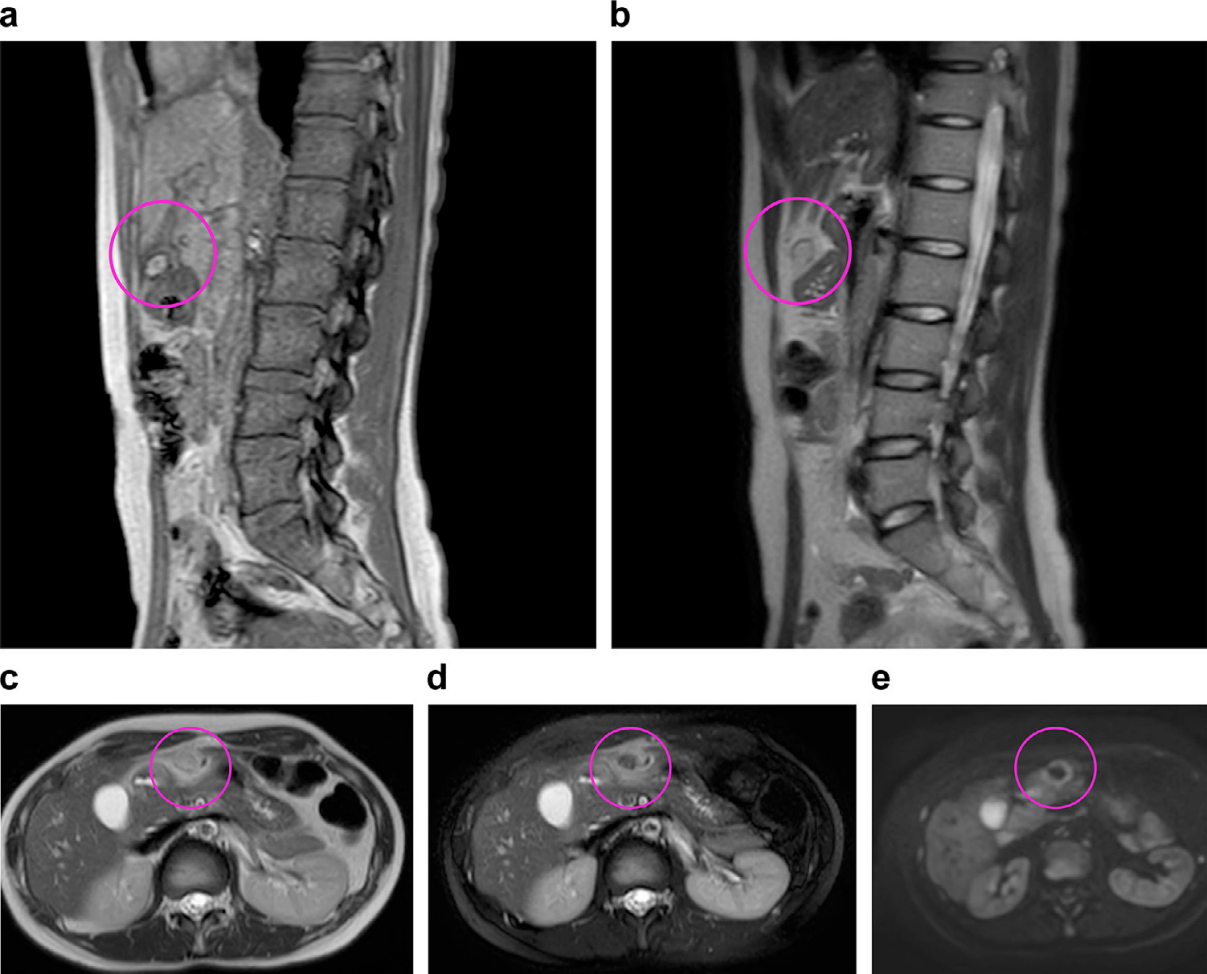
Magnetic resonance imaging findings in Case 2. (A) T1-weighted imaging, (B) T2-weighted imaging, (C) T2-weighted imaging, (D) fat-suppressed T2-weighted imaging, (E) Diffusion-weighted imaging. T1-weighted imaging shows a 1-cm oval mass with high signal on the right side of the hepatic round ligament (A) (circle). The mass is comparable with the surrounding fat on T2-weighted imaging (B, C) and is hypointense with surrounding high signal area on fat-suppressed T2-weighted imaging (D). Diffusion-weighted imaging shows high signal only on the margins of the mass (E).

**Case 2:** A 28-year-old woman in the fourth week of pregnancy came to our hospital with a chief complaint of persistent severe postprandial epigastric pain for 5 days. Her BMI was 19.5 kg/m^2^. Blood and urine tests done 4 days before, at a local clinic, showed no abnormalities. Two days before, ultrasound done at another general hospital did not show obvious abnormal findings. Palpation revealed localized tenderness on the epigastric area without guarding. Upper gastrointestinal endoscopy showed no abnormal findings. Blood tests revealed a WBC count of 9250/ μL and a CRP of 0.57 mg/dL. For the ultrasound examinations, we used Aplio XG (Canon Medical Systems, Tochigi, Japan) with a 12L5 linear probe set at 8-MHz frequency. Ultrasound revealed an oval-shaped hyperechoic mass, which had a hypoechoic rim and was adjacent to the falciform ligament, and inflammation of the surrounding fat (Fig. 3). Color Doppler showed no blood flow signal inside the mass, possibly reflecting infarction. These ultrasound findings suggested ischemic or inflammatory changes in the fatty appendage that was contiguous with the falciform ligament. On the same day, MRI showed a 1-cm oval mass with T1-weighted image (T1WI) hyperintensity on the right side of the hepatic round ligament (Fig. 4). On the fat-suppressed T2-weighted image, the mass was hypointense, but the rim and surrounding area were hyperintense, which suggest inflammatory and edematous changes. The T2-weighted image (T2WI) internal characteristics of the mass suggested the presence of fatty tissue and led to a suspicion of localized panniculitis or fat necrosis. Based on a diagnosis of FLA, the patient was prescribed with analgesics and antibiotics and placed on observation. At a follow-up visit one week later, the symptoms had resolved and repeat ultrasound showed shrinkage of the hyperechoic mass and decreased echogenicity of the surrounding fat. Therefore, we decided to conclude the final medical consultation.

## Discussion

FLA is very rare and has little evidence on its clinical and imaging findings. In this report, the differential diagnoses in both patients who had localized epigastric pain included acute gastritis, gastric ulcer, cholecystitis, pancreatitis, and many others. Therefore, it is difficult to assume FLA based on clinical findings alone. To our knowledge, only 24 cases of FLA, including FLAT [2], had been reported, but this number has increased rapidly in recent years because of improved diagnostic imaging capabilities. The present cases showed edematous/inflammatory changes in the fatty appendage contiguous with the falciform ligament and were presumed to be similar to FLAT. FLA is an ischemic/inflammatory change of the fatty appendage contiguous with the falciform ligament and is often considered to be similar to IFFI in terms of clinical and imaging findings, particularly to epiploic appendagitis, which has both hemodynamic and nonhemodynamic forms. Similarly, FLA is considered to have 2 types: (1) circulatory disturbance secondary to torsion, infarction, or direct compression of the appendage and (2) inflammatory spillover from the falciform and round ligaments of the liver. Inflammatory spillover rarely causes FLA, but a case of FLA secondary to Roux-en-Y bypass surgery has been reported [3].

The few available reports on FLA may have 3 reasons: (1) the disease itself is rare, (2) it may have been missed on an ultrasound, and (3) it resolves spontaneously and patients do not return to the hospital. Among the studies on FLA, the most common imaging reported to have been used were ultrasound and CT [1,^2^,4]. In this report, the ultrasound images of the 2 cases were consistent with the location of the epigastric tenderness and characterized by the following 3 features: (1) contiguous with the falciform and round ligaments of the liver; (2) hyperechoic oval mass without Doppler signals, and the margins may be hypoechoic depending on the degree and course of ischemia; and (3) increased echogenicity of the surrounding fatty tissue because of the spread of inflammation. When these 3 findings are present, a diagnosis of FLA can be made.

**Fig. 5:**
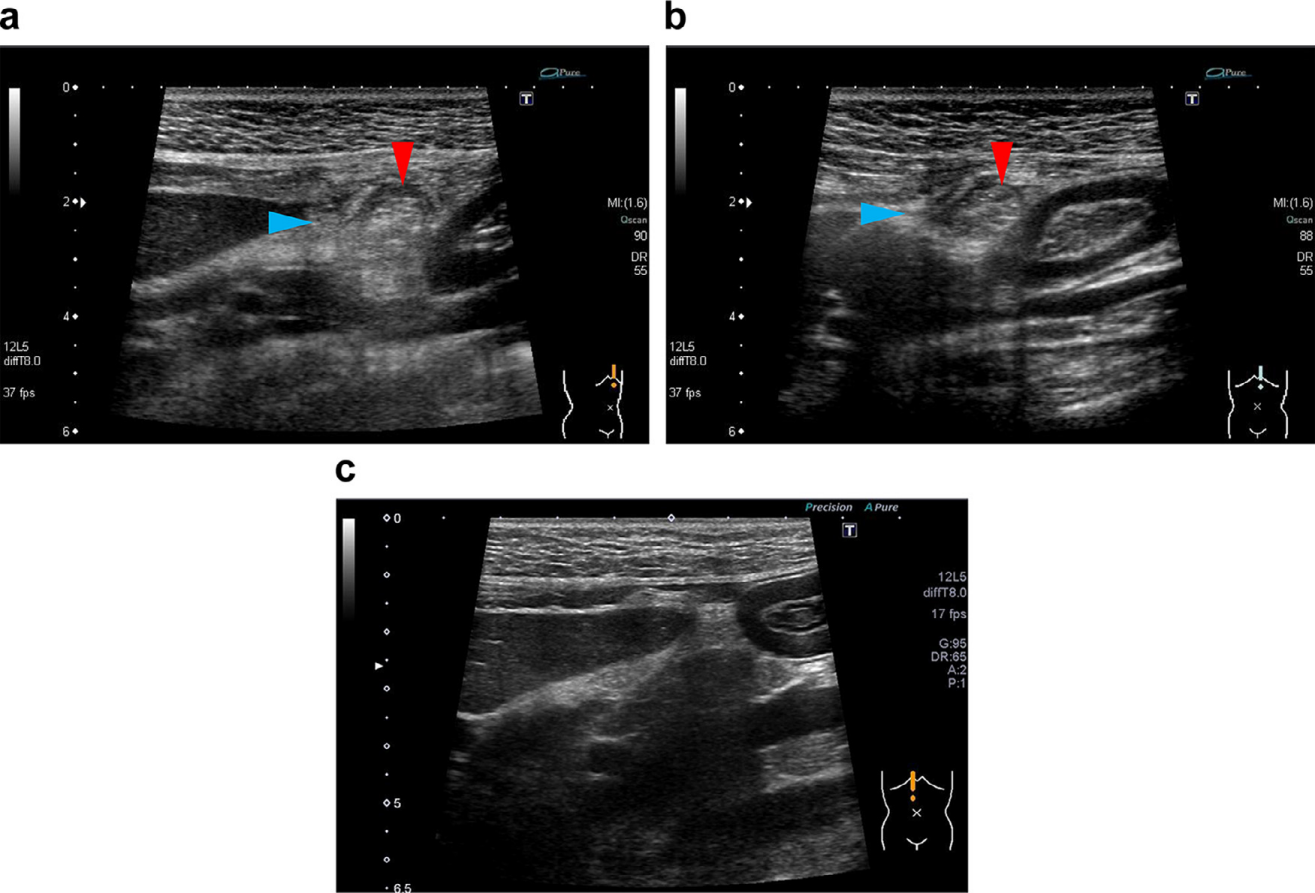
Changes in the ultrasound findings of falciform ligament appendagitis on follow-up of Case 2. (A) At the time of onset, there is an oval hyperechoic mass with a peripheral hypoechoic rim (red arrow head) and increased echogenicity of surrounding fat (blue arrow head). (B) One week later, the mass has shrunk remarkably (red arrow head) and the surrounding inflammatory fat is reduced (blue arrow head). (C) Eight months later, there is complete resolution of the hyperechoic mass and surrounding inflammatory fat.

Given the anatomic location, a lesion in the falciform ligament is located at a close distance from the ultrasound probe; therefore, the use of a regular 3.5-MHz convex probe alone would make it difficult to notice the lesion. Given that both the patients were slightly leaner than standard body weight, we were able to use a high-frequency linear probe set at 8-10-MHz frequency. For mildly obese patients in whom this linear probe cannot be used, we recommend the 6-MHz high frequency convex probe. Thus, when FLA is suspected, the use of a high-frequency probe may improve diagnostic performance. In case 2, follow-up ultrasound demonstrated gradual shrinkage of the hyperechoic mass and reduction of the surrounding inflammatory fat until complete resolution (Fig. 5). There had been only one report on a case that was followed up on ultrasound [5]. Our case had an imaging course similar to that of this case report; we consider that the fatty appendage underwent progressive necrosis due to torsion and was eventually amputated from the ligament.

CT classically demonstrates FLA as an area of fat density with a thin peripheral rim of hyperattenuation, with or without the classical central dot corresponding to a thrombosed vein [6,7]. In case 1, the additional contrast-enhanced CT (5 mm slice, transverse view only) did not clearly show the oval mass seen on ultrasound. This may be explained by the fact that the inflammation was minimal and the size of the mass was small to be detected on 5-mm-slice images. Nevertheless, the inflammatory changes along the hepatic round ligament were definitely present. These CT finding and the clear oval fatty mass on ultrasound suggested inflammatory changes of the fatty appendage.

The diagnosis of infarcted ligamentum teres hepatis lipoma by MRI has only been reported in one case [8]. In case 2, MRI showed an oval mass that had a high signal on T1WI, was in contact with the hepatic round ligament, had a T2WI signal equivalent to the surrounding fatty tissue, and fat-suppressed T2WI low signal. These findings strongly suggest that the oval mass is a fatty mass. Furthermore, fat-suppressed T2WI showed a high signal area around the mass, which may be changes that suggest edema/inflammation of the fatty mass. This is the first report of detailed MRI findings using T1WI, T2WI, fat-suppressed T2WI, and diffusion MRI for the diagnosis of FLA.

A few cases of surgical treatment for FLAT had been reported [2,9]. Because most cases of FLA are known to heal without surgery [1,7], conservative treatment with rest and nonsteroidal anti-inflammatory drugs was considered first-line treatment in our cases, which showed spontaneous resolution of symptoms. Ultrasound is often the first imaging test performed for acute abdomen, and proper diagnosis of FLA can prevent unnecessary invasive treatment. Therefore, it is important to understand the ultrasound characteristics of FLA and repeat real-time observation in response to symptoms for appropriate management.

## Patient consent

Written informed consent was procured from the patients for publication of this case.

## Author contributions

Ultrasound interpretation and manuscript writing: DM, CT and MRI interpretation: MS, and clinical diagnosis: YF

## Ethics approval

The Fukuoka Tokushukai Medical Center Research Ethics Committee has confirmed that no ethics approval was required. All procedures were in accordance with the ethical standards of the responsible institutional and national committee on human experimentation and with the Helsinki Declaration of 1975 and its late amendments.

## Data availability

Data sharing not applicable to this article as no datasets were generated or analyzed during the current study.

## Code availability (software application or custom code)

Not applicable.

## Appendix Supplementary materials

Image, application 1
